# IsoCleft Finder – a web-based tool for the detection and analysis of protein binding-site geometric and chemical similarities

**DOI:** 10.12688/f1000research.2-117.v2

**Published:** 2013-05-02

**Authors:** Natalja Kurbatova, Matthieu Chartier, María Inés Zylber, Rafael Najmanovich

**Affiliations:** 1 European Bioinformatics Institute, Wellcome Trust Genome Campus, Cambridge, CB10 1SD, UK; 2 Department of Biochemistry, Université de Sherbrooke, Sherbrooke, J1H 5N4, Canada

## Abstract

IsoCleft Finder is a web-based tool for the detection of local geometric and chemical similarities between potential small-molecule binding cavities and a non-redundant dataset of ligand-bound known small-molecule binding-sites. The non-redundant dataset developed as part of this study is composed of 7339 entries representing unique Pfam/PDB-ligand (hetero group code) combinations with known levels of cognate ligand similarity. The query cavity can be uploaded by the user or detected automatically by the system using existing PDB entries as well as user-provided structures in PDB format. In all cases, the user can refine the definition of the cavity interactively via a browser-based Jmol 3D molecular visualization interface. Furthermore, users can restrict the search to a subset of the dataset using a cognate-similarity threshold. Local structural similarities are detected using the IsoCleft software and ranked according to two criteria (number of atoms in common and Tanimoto score of local structural similarity) and the associated Z-score and p-value measures of statistical significance. The results, including predicted ligands, target proteins, similarity scores, number of atoms in common, etc., are shown in a powerful interactive graphical interface. This interface permits the visualization of target ligands superimposed on the query cavity and additionally provides a table of pairwise ligand topological similarities. Similarities between top scoring ligands serve as an additional tool to judge the quality of the results obtained. We present several examples where IsoCleft Finder provides useful functional information. IsoCleft Finder results are complementary to existing approaches for the prediction of protein function from structure, rational drug design and x-ray crystallography. IsoCleft Finder can be found at: http://bcb.med.usherbrooke.ca/isocleftfinder.

## Introduction

Current computational methods for the prediction of protein function from structure are restricted to the transfer of functional annotation based on similarities. In instances where global sequence or structural similarities do not provide clues about protein function, one alternative is to detect binding-site similarities. Such similarities may help predict potential small-molecules that bind to the protein, thus providing valuable functional information. Binding-site similarities may also help recognize atoms involved in non-bonded interactions thus aiding in the rational design of inhibitors.

Methods for the detection of local structural similarities vary primarily in the type of representation (generally simplified) and search method, usually via the detection of sub-graph isomorphism ^1^ or geometric hashing ^2^. However, due to the time-consuming nature of the detection of sub-graph isomorphism, methods have resorted mostly to simplifications in the form of pseudo-atoms ^3, 4^ or simplified surface patches ^5^. Shulman-Peleg *et al.* ^6, 7^ combined the simplified representation, graph-matching-based method of Schmitt *et al.* ^4^ with a geometric hashing pre-screening step. With the exception of IsoCleft ^8^, methods that make use of full atomic representation, i.e. utilizing the coordinates of all non-hydrogen atoms, are few and only applicable in limited cases. The method of Kobayashi & Go ^9^ requires the superimposition of bound ligands. Brakoulias & Jackson ^10^ use a geometric method to compare a large dataset of molecular environments of phosphate groups that also require the pre-definition of the molecular environments from the position of the PO _4_ groups. A more thorough review of methods for the detection of local structural similarities can be found in Najmanovich *et al.* ^11^ and references therein. The IsoCleft program, developed for the detection of binding-site 3D local atomic similarities ^8^ uses an all atom (non-simplified) representation, in a two-stage graph-matching process. IsoCleft can compare large binding sites in a timely manner and does not need any information regarding bound ligands.

### IsoCleft

This section recapitulates the description of the IsoCleft program, the engine behind the IsoCleft Finder tool, as presented previously ^8^. IsoCleft utilizes graphs to conceptually represent binding-sites in order to take advantage of the well-developed graph-matching techniques for the detection of similarities between graphs (sub-graph isomorphism). A graph is defined by a set of nodes and edges where each edge connects a pair of nodes through a specific property. Graphs can be used to represent any entity (real objects, ideas, etc.) composed of smaller parts (nodes) and their (pairwise) relationships to each other (edges). Given two sets of atoms defining the query cleft and a target binding-site under comparison, the question that needs to be answered is: what is the largest subset of atoms in both clefts in direct correspondence with each other geometrically as well as chemically?

In the representation of a binding site as a graph, nodes represent atoms *A _i_^m^* (atom *i* out of a total of *N _m_* in molecule *m*) while an edge represents the Euclidian distance between any two atoms. In other words, in this representation of a binding-site all atoms define nodes and all nodes are interconnected by edges. Both nodes and edges are assigned properties (referred as colorings in graph theory). Nodes (atoms) are assigned an atom type property that determines the types of molecular interactions they may participate in. IsoCleft employs the atom type scheme of Sobolev *et al.* ^12^, in which atoms are classified into eight types: hydrophilic, hydrogen bond (HB) acceptor, HB donor, hydrophobic, aromatic, neutral, neutral-donor and neutral-acceptor. Edges are assigned a (coloring) value *d _c_*, the Euclidean distance between the atoms they connect in the 3D binding site structure. Viewing each cleft as a graph, the task is to find the largest common sub-graph isomorphism. This is done through the construction of a second graph, known as an association or correspondence graph, derived from the two initial cleft graphs under comparison.

The construction of the association graph requires the definition of nodes and edges. Nodes represent chemical similarity while edges represent geometric similarity. Each node *k* = { *i,t*} defines a possible correspondence between a pair of atoms *A _i_^m^* and *A _t_^n^* of identical type, one from each cleft under comparison (clefts *m* and *n*). This condition assures that the final subset of atoms in common between the two clefts corresponds pairwise to the same type. An association graph edge is created between two association graph nodes if the binding-site graph edges connecting the corresponding pairs of atoms in each binding-site graph are similar. In other words, if we have two nodes *k* = { *i,t*} and *l* = { *j,s*} in the association graph, an edge will be created if:

$$|d_{c}(i,j)-d_{c}(t,s)|\leq D_{node}\mathrm{(1)}$$

The above condition of geometric similarity, used when creating edges in the association graph, is such that a clique (a subset of nodes fully connected by edges) corresponds to a subset of atoms in each cleft in which all pairwise distances between atoms in one cleft are satisfied by the corresponding atoms in the other cleft. The parameter *D _node_* implicitly permits to accommodate the effect side-chain flexibility to a certain extent. In IsoCleft Finder, this parameter is set at *D _node_* = 4 Å.

**Figure 1.**
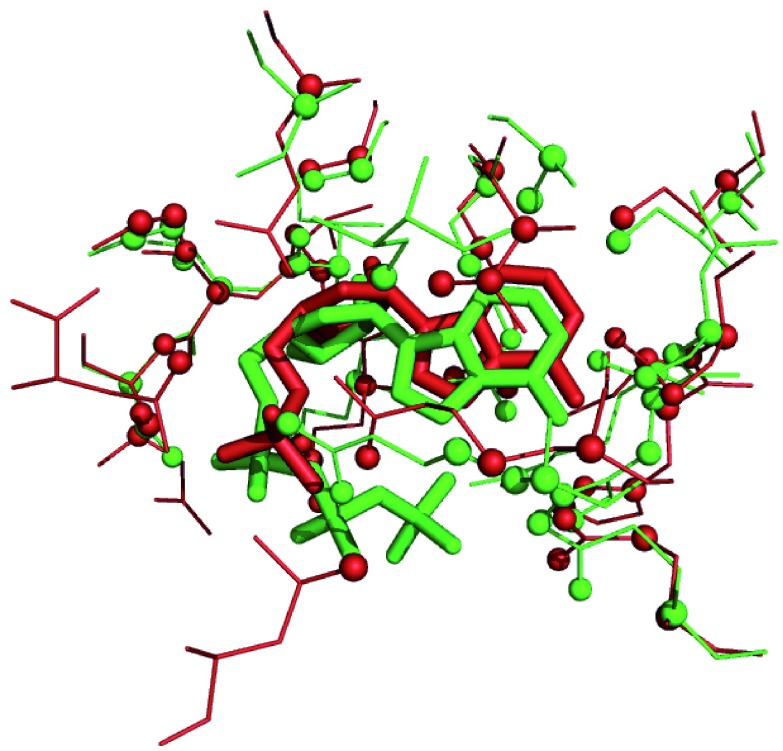
Superimposition of *E. coli* aspargine synthase (PDB code 12as) bound to adenosine monophosphate (AMP, in red) and aspartyl t-RNA synthase (PDB code 1b8a) from *Thermococcus kodakaraensis* bound to adenosine-5´-triphosphate (ATP, in green) sharing 27% sequence identity. Detected common binding-site atoms are displayed as spheres. While the superimposition was performed using binding-site atoms, the RMSD of the equivalent ligand atoms is 1.1 Å.

The objective of the graph matching procedure is the detection of the largest clique, i.e. the largest subset of atoms of identical type in equivalent spatial positions between the two clefts. This set of atoms can be used to superimpose both clefts under comparison ( Figure 1).

The combinatorial nature of association graphs can lead to exponentially large graphs, both in terms of number of nodes and density of edges. This is a major drawback when employing association graphs to detect common sub-graph isomorphisms since the computational cost of clique detection algorithms increases very rapidly with the size of the association graph. IsoCleft introduces two innovations that allow us to overcome this common problem associated with graph matching.

The first innovation is to perform the graph matching in two stages. In the first stage, an initial superimposition of the two binding-sites under comparison is performed via the detection of the largest clique in an association graph constructed using only C _α_ atoms of equivalent residues in the two clefts. The minimum rank-order ( *r*) of each residue's JTT substitution matrix probabilities ^13^ is used to set the level of allowed residue similarity (association graph node coloring). Edges in the first-stage association graph represent, as described above, Euclidean distance differences ( Equation 1) but this time between C _α_ atoms, with *D _node_* = 3.5 Å. Once the largest C _α_ clique is obtained, its transformation matrix and translation vectors are used to superimpose all atoms in both clefts using the least square method of Arun *et al.* ^14^. In other words, the first stage performs a C _α_-based superimposition of all atoms in the clefts under comparison based on the detection of the largest subset of similar residues in equivalent spatial positions. The residue-similarity JTT minimum rank-order threshold parameter is set at *r* = 5.

In the second graph matching stage, all non-hydrogen atoms are used. Association graph nodes are created with the requirement that two atoms, one from each cleft, be of the same type, as described earlier, and that their spatial distance after the first stage superimposition be within the default value *n* = 4 Å. This distance threshold is used to decrease the size of the association graph and it is the reason why the initial graph matching stage is performed. In effect, a pair of atoms, one from each cleft and of identical type that would otherwise define an association graph node, will be too distant to do so after the first-stage superimposition. The Ca atoms artificially included in the set of cleft atoms for the first stage are not considered in the second stage and thus do not contribute directly to the detection or measurement of similarity. IsoCleft utilizes the Bron & Kerbosch algorithm ^15^ to detect the largest clique in the association graphs on both stages of the graph matching process.

The second innovation introduced in IsoCleft is to exploit the fact, noted by Bron & Kerbosch ^15^, that their algorithm has the tendency to produce the cliques in decreasing size order. IsoCleft implements what we call “Approximate Bron & Kerbosch” (ABK). In ABK, the first clique is selected as the solution (and the search procedure is stopped) rather than detecting all cliques in order to find the largest. Utilization of ABK allows us to obtain an optimal or nearly optimal solution in a fraction of the time that would be needed using the original algorithm, without any noticeable effect on the results. The fractional loss in accuracy when using ABK compared to the original Bron & Kerbosch algorithm is likely to be minor compared to the effect of intrinsic noise inherent to biological systems (in part as a consequence of flexibility) in addition to the noise introduced with the choice of empirical parameters (e.g., in the definition of the association graph).

The stand-alone version of the IsoCleft program (freely available for academic users upon request) requires a number of user-defined parameters. In IsoCleft Finder, these parameters are fixed with default values previously obtained through an extensive heuristic search in parameter space. The default values maximize the average area under the Receiver-Operator Characteristic (ROC) curve (AUC) ^8^ in predictions of ligand-binding classes based on binding-site similarities across non-homologous protein families that convergently evolved to bind similar ligands.

## Results

This article describes: 1. The implementation of the non-redundant dataset of target binding-sites with known levels of cognate similarity (ICFDB v. 1.6), 2. The IsoCleft Finder web-interface for the IsoCleft program with which to determine the level of binding-site similarity between query binding-sites and those in the ICFDB 1.6 dataset, 3. A set of visual and computational post-search analysis tools that help judge the quality of the detected hits, and 4. The results for several cases in which IsoCleft Finder provides useful functional information. All together, IsoCleft Finder is a unique web-based tool that provides direct and convenient access to the detection of atomic level binding-site similarities to research groups without bioinformatics expertise or the necessary computational resources required for large-scale database searches.

### Target binding-site dataset

A non-redundant dataset of target binding-sites, called ICFDB (current version 1.6), was developed based on the Procognate database ^16^. Procognate provides cognate-similarity levels for a large subset of proteins in the PDB database. A cognate ligand is a natural ligand, that is, a substrate or cofactor of the given protein (as recorded in the KEGG database ^17^). In other words, in a complex between a protein and a cognate ligand, the structural determinants of molecular recognition were subjected to natural selection. While statistically significant binding-site similarities are interesting irrespective of what ligand the target protein is bound to, if that ligand is highly similar to a cognate ligand, these similarities are more informative regarding the potential biological function of the query protein. Cognate similarities in Procognate can vary from zero, where there is no similarity between the bound ligand and the cognate ligands, to one, when a cognate ligand is the one present in the solved structure. The choice of a cognate similarity threshold is subjective and it is therefore impossible to determine a single particular threshold appropriate for all purposes and query types as the number of atoms in the ligand has an effect on this threshold. A statistically relevant hit to a binding site bound to a large ligand with smaller cognate similarity might offer equal or greater relevant functional clues as a hit to a smaller ligand with greater cognate similarity.

**Figure 2.**
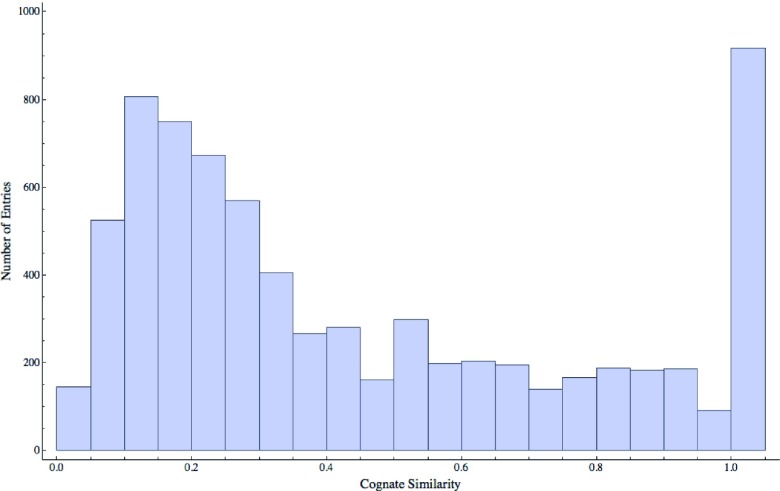
Distribution of cognate similarity in the IsoCleft Finder dataset of target binding-sites. The majority of ligands display low cognate similarity. The peak at the extreme right of the distribution represents those cases where the bound ligands are nearly identical to the cognate ligands.

Our dataset contains a subset of unique Pfam ^18^ ligand combinations from Procognate with associated levels of cognate-ligand similarity. The dataset contains 7339 entries of which 970 have a coefficient of cognate-ligand similarity equal or larger than 0.95 ( Figure 2). While any statistically relevant match may offer potential clues regarding the function of a protein, matches to complexes involving ligands with high levels of cognate similarity may in addition reflect distant evolutionary relationships. The dataset contains 6110 unique PDB entries (with resolution 2.16 ± 0.45 Å and 27 NMR structures), representing 6093 different Uniprot entries ^19^ belonging to 997 Pfam families and containing 29905 unique gene ontology (GO) molecular function terms ^20^. In terms of ligands, ICFDB v. 1.6 contains 3833 different small-molecules. The dataset is available for browsing at http://bcb.med.usherbrooke.ca/files/ICFDB1_6.txt, can be downloaded as a zip file through a link in the IsoCleft Finder web page http://bcb.med.usherbrooke.ca/isocleftfinder or downloaded below.

IsoCleft Finder Dataset version 1.6 (ICFDB v. 1.6) Non-redundant dataset of 7339 entries representing unique Pfam/PDB-ligand (hetero group code) combinations with known levels of cognate ligand similarity. Each entry includes a ligand and a set of interacting residues in PDB format.

### Web-interface

The IsoCleft Finder tool consists of three pages: input, cleft definition and output. Help is provided in the form of notes and hovering tips.

**Figure 3.**
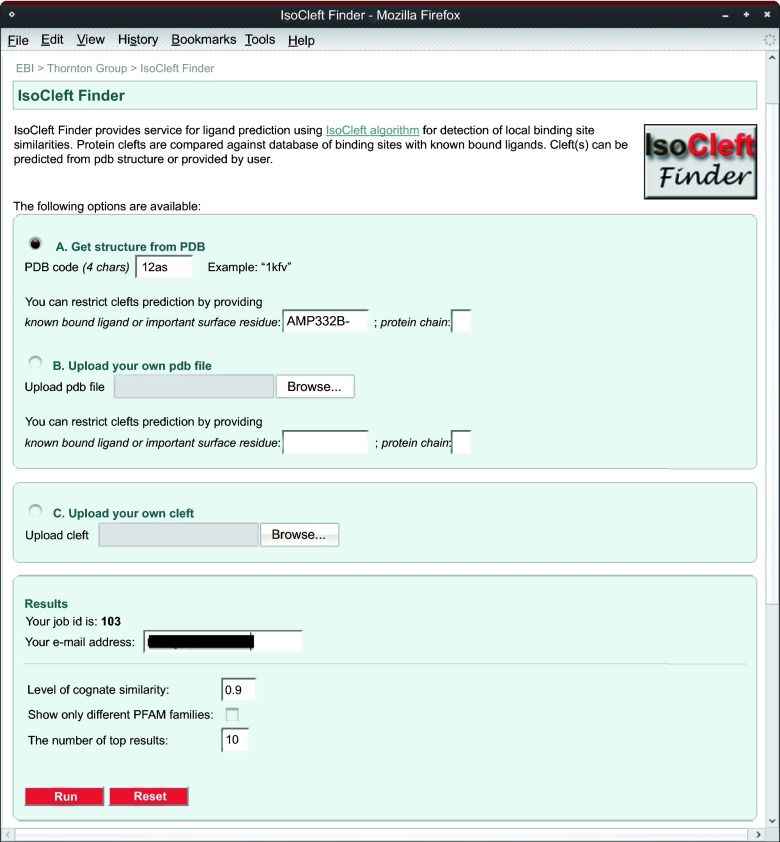
IsoCleft Finder input page showing the different options to upload or define the query cleft.

The input page ( Figure 3) allows a user to provide the 4-letter code of an existing PDB entry or upload a file in PDB format containing an entire structure or only the atoms defining a cleft. A cleft file must contain four or more atoms and include the alpha carbon atom of any residues represented. When an entire structure is used, the three largest clefts are defined automatically using our own implementation of the Surfnet algorithm ^21^. Furthermore, the user may provide some parameters to better define the clefts, such as a chain or residue/bound-ligand identifier. The former avoids clefts defined at the interface between chains while the latter helps detect a cleft that is not among the largest three. To identify a residue or bound ligand we utilize the residue ID in RESNUMCA format, where RES represents the three-letter PDB residue code, NUM represents the residue number, C represents the chain identifier and A represents the alternative location code. A dash is used in cases where C and/or A are blank characters in the coordinates file (e.g., BTN300-- or ASN94A-). If, for example, only the RES code is given (particularly useful in the case of ligands), we utilize a basic pattern matching procedure to detect all possible residue ID matches within the PDB file and the user then selects the appropriate match to proceed. The user may provide an email address, in which case a link with the results is sent via email. Otherwise, the user must save the link of the results page upon job submission or leave the browser window open.

The input page offers the possibility to output the top hits belonging to different Pfam families. This option permits the inspection of the results from a broader perspective from the protein point of view in the sense that high scoring matches of similar binding-sites will in effect represent cases of divergent evolution with more remote homologues or cases of convergent evolution.

Finally, a threshold value for the minimum level of cognate similarity can be chosen. Higher values decrease the size of the target dataset as per the distribution in Figure 2.

**Figure 4.**
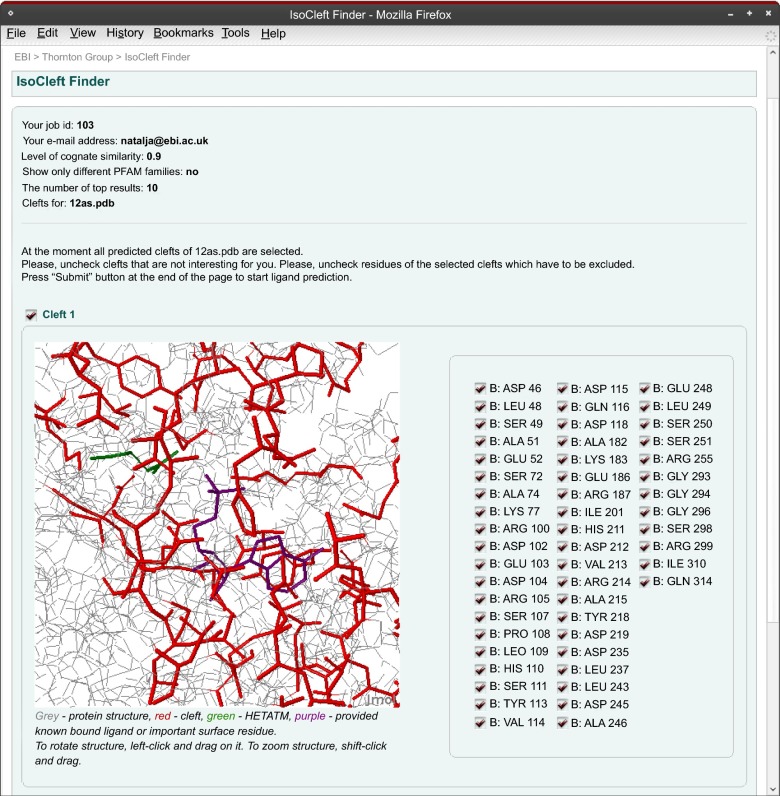
Refinement of cleft definition. The user is able to de-select residues initially included in the cleft(s).

The cleft definition page ( Figure 4) contains consecutive panels, one for each cleft. The user may deselect those clefts that are not relevant. Each cleft is shown in an interactive 3D Jmol ( http://www.jmol.org) applet. All residues that contribute at least one atom to the cleft are shown in red. The right panel can be used interactively to deselect any unnecessary residues. Any ligands present in the cleft are also colored.

Running times vary depending on input values and system load. In particular, the threshold level of cognate similarity defines what subset of the binding-site target dataset is used in the search and affects considerably the running time as, for example, with a value of 0.95 only 970 pairwise comparisons will be required while a value of 0.0 will imply 7339 pairwise comparisons.

### Post-search analysis tools

**Figure 5.**
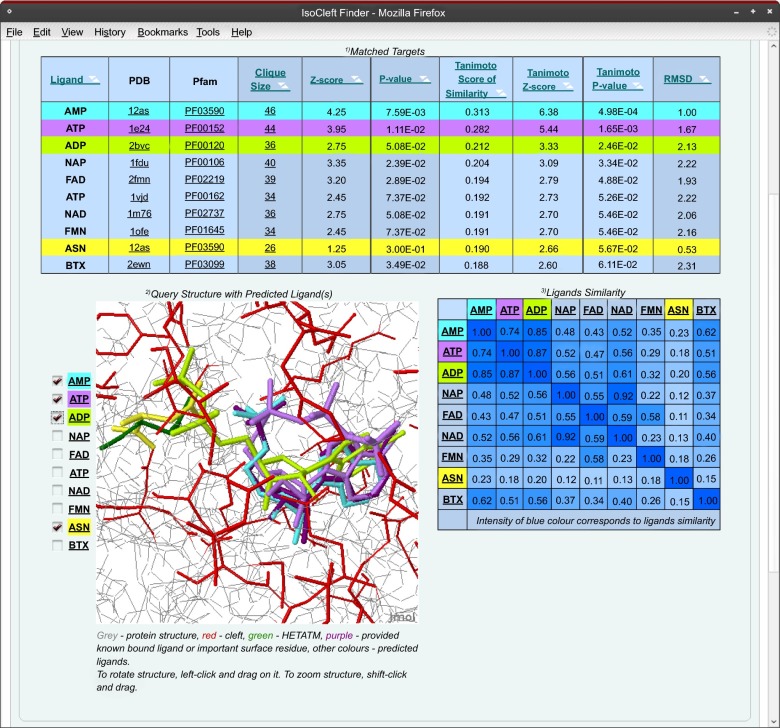
Example of the IsoCleft Finder output containing the list of hits (top) superimposed on the query binding-site of 12as (bottom left) and the target ligand similarities (bottom right). See http://bit.ly/Xz0Qm.

The output page ( Figure 5) returns the top scoring (most similar) binding-sites in the form of a table as well as a Jmol applet in which the ligands bound to the target binding-sites are superimposed on the query binding-site based on the detected atomic similarities. For each hit, the number of atoms in common ( *N _C_*) is presented as well as a Tanimoto Score of Similarity ^8^:

$$T=N_{C}/(N_{A}+N_{B}-N_{C}) (2)$$

where *N _A_* and *N _B_* represent the number of atoms in the query and target binding-sites, respectively. Two measures of statistical significance, Z-score and p-value, are calculated for the number of atoms in common and the Tanimoto Score of Similarity. The mean (µ) and standard deviation (σ) values are calculated and used to define the Z-score, *z* = (T–µ)/σ. For the purpose of calculating p-values, data points with *z* < –3 or *z* > 7 are removed followed by recalculation of µ and σ. This process is repeated up to five times. The p-value is derived from the final Z-scores using an extreme value distribution as follows ^22^:

p=1−exp⁡(−e−zπ/6−Γ'(1))   (3)

where Γ is the gamma function and Γ’(1) = –0.5772157.

It is important to note that as the size of the target binding-sites varies, one may find hits that when compared to each other contain larger numbers of atoms in common to the query but smaller Tanimoto Scores of Similarity. Several such cases can be found in Table 1– Table 3. For a given number of atoms in common, as the size of a target binding-site increases, the statistical significance of its Tanimoto Score of Similarity decreases. As it is not possible to decide if a large number of atoms in common is more desirable than a large fraction of atoms in common, both measures should be used to determine the functional relevance of the hits obtainer.

**Table 1.** IsoCleft Finder results for 3e7e sorted in decreasing order of Tanimoto Score of Similarity.

Target		Atoms in Common			Similarity		
PDB	Ligand	N _c_^1^	Z-score	p-value	TSS ^2^	Z-score	p-value
1l3r	ADP	47	3.63	1.68E-02	0.144	4.37	6.51E-03
1gy3	ATP	45	3.37	2.33E-02	0.138	4.08	9.44E-03
2ijm	ADP	44	3.24	2.74E-02	0.138	4.08	9.44E-03
3dqw	SAP	41	2.86	4.47E-02	0.127	3.55	1.86E-02
1iah	ADP	40	2.73	5.25E-02	0.119	3.16	3.04E-02
3b2t	M33	38	2.47	7.24E-02	0.118	3.11	3.24E-02
2aqx	ATP	39	2.60	6.17E-02	0.116	3.02	3.66E-02
1d7l	RFL	45	3.37	2.33E-02	0.115	2.97	3.89E-02
2bl4	NAD	41	2.86	4.47E-02	0.109	2.68	5.60E-02
2nu6	COA	40	2.73	5.25E-02	0.105	2.48	7.12E-02

^1^ Number of atoms in common.

^2^ Tanimoto Score of Similarity.

**Table 2.** IsoCleft Finder results for 2re3 sorted in decreasing order of Tanimoto Score of Similarity.

Target		Atoms in Common			Similarity		
PDB	Ligand	N _c_^1^	Z-score	p-value	TSS ^2^	Z-score	p-value
1pwh	PLV	37	3.25	2.73E-02	0.257	3.39	2.29E-02
2amq	3IH	39	3.59	1.77E-02	0.235	2.77	4.95E-02
1dm3	ACO	36	3.08	3.39E-02	0.232	2.69	5.49E-02
1hwy	NAD	36	3.08	3.39E-02	0.220	2.36	8.29E-02
1q6p	213	35	2.90	4.20E-02	0.213	2.16	1.05E-01
1dqa	NAP	36	3.08	3.39E-02	0.211	2.11	1.12E-01
2vfs	FAD	35	2.90	4.20E-02	0.183	1.33	2.77E-01
1gpe	FAD	36	3.08	3.39E-02	0.182	1.30	2.85E-01
1v0j	FAD	36	3.08	3.39E-02	0.178	1.19	3.31E-01
1coy	FAD	35	2.90	4.20E-02	0.178	1.19	3.21E-01

^1^ Number of atoms in common.

^2^ Tanimoto Score of Similarity.

**Table 3.** IsoCleft Finder results for 2re9 sorted in decreasing order of Tanimoto Score of Similarity.

Target		Atoms in Common			Similarity		
PDB	Ligand	N _c_^1^	Z-score	p-value	TSS ^2^	Z-score	p-value
1tzf	C5G	33	3.08	3.39E-02	0.262	3.27	2.66E-02
2ov2	GCP	33	3.08	3.39E-02	0.248	2.89	4.28E-02
1zpd	DPX	33	3.08	3.39E-02	0.246	2.84	4.58E-02
1n78	GOM	33	3.08	3.39E-02	0.232	2.46	7.32E-02
1suq	185	33	3.08	3.39E-02	0.226	2.30	8.92E-02
1u8x	NAD	33	3.08	3.39E-02	0.198	1.55	2.18E-01
1q83	TZ5	35	3.46	2.09E-02	0.193	1.41	2.53E-01
1gge	HDD	33	3.08	3.39E-02	0.192	1.38	2.61E-01
2p6e	MYA	33	3.08	3.39E-02	0.163	0.60	5.60E-01
1eex	COY	35	3.46	2.09E-02	0.161	0.55	5.85E-01
1k7y	B12	34	3.27	2.66E-02	0.160	0.52	5.98E-01

^1^ Number of atoms in common.

^2^ Tanimoto Score of Similarity.

Several links are provided to download the results and to access further information regarding the matches. These include links to the list of atomic correspondences and superimposed coordinates as well as to external sources such as Pfam ^18^ and PDBsum ^23^.

Finally, a second table shows the pairwise topological similarity ^24^ between the target ligands present in the top scoring target binding-sites. Similarities between top scoring ligands represent an independent source of evidence in support of the biological relevance of the detected binding-site similarities. A large number of equivalent ligand atoms in equivalent positions in space (as a consequence of the binding-site superposition produced by IsoCleft) may point to binding-site atoms in the query protein that are important from a molecular recognition point of view ^8^. In the case shown in Figure 5, we used the adenosine monophosphate (AMP) bound cleft of *Escherichia coli* aspargine synthase (PDB code 12as) as query. L-aspargine (ANS), the product of the reaction, is also bound. The bound AMP and ASN molecules define two independent binding-sites, both of which are found within the top scoring hits. The second and third hits (ATP and ADP) represent different Pfam families and folds but, as can be seen in Figure 5, their adenine and ribose moieties superimpose very well compared to the bound AMP molecule.

**Figure 6.**
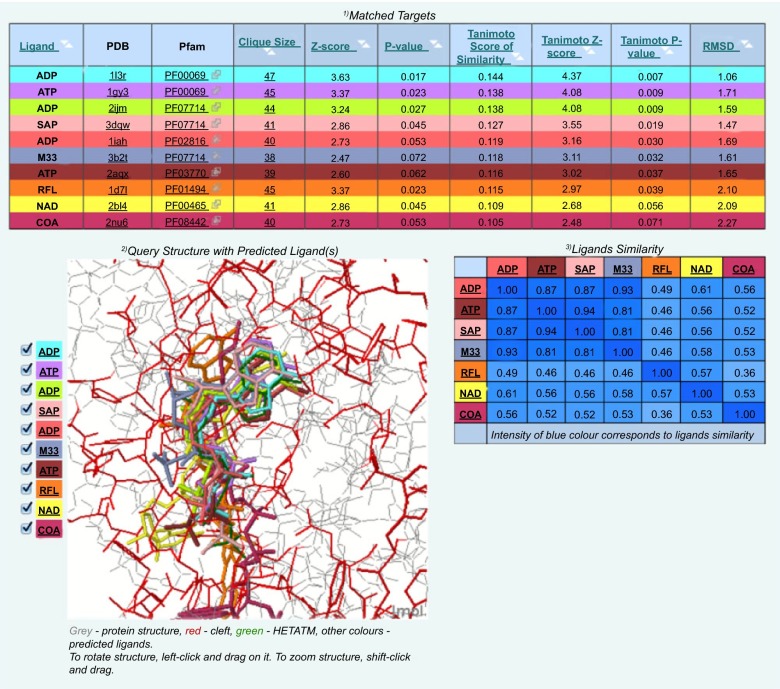
Example of the IsoCleft Finder output containing the list of hits (top) superimposed on the query binding-site of 3e7e (bottom left) and the target ligand similarities (bottom right).

**Figure 7.**
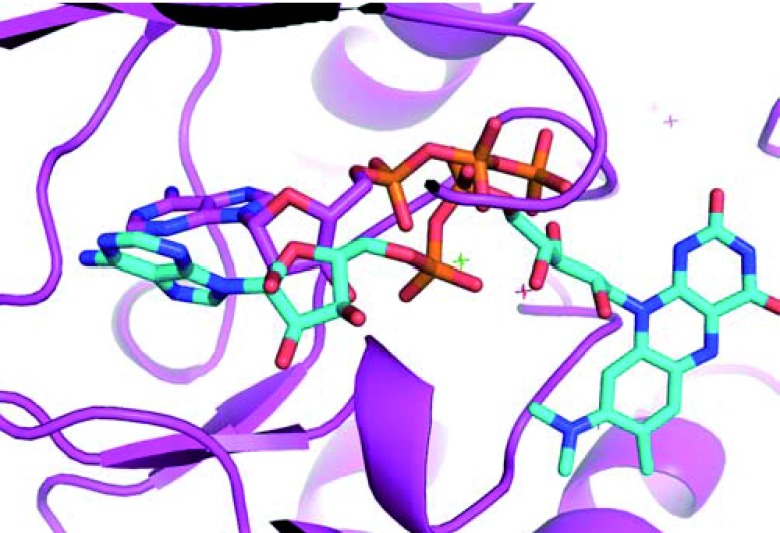
Protein Bub1 (PDB code 3e7e) bound to ATP (in magenta). The riboflavin (RFL) ligand from PDB entry 1d7l (in cyan) is superimposed based on the similarities identified by IsoCleft. This result suggests that the two proteins have a similar arrangement or residues that were conserved throughout evolution to bind the common moieties of ATP and RFL.

The strength of the IsoCleft and the IsoCleft Finder interface can be seen with the Human mitotic spindle checkpoint kinase Bub1 (PDB code 3e7e) query protein in Figure 6. Among the found hits ( Table 1), the six top hits represent protein kinase (PF00069), tyrosine kinase (PF07714) and alpha-kinase (PF02816) Pfam families. The seventh hit is PDB code 2aqx, a phosphotransferase (inositol polyphosphate-PF03770) that has a distinct domain fold from the typical kinase domain, but one that has independently evolved to bind ATP. The eighth hit is PDB code 1d7l (FAD binding domain-PF01494) bound to riboflavin (RFL). The superimposition of the ligands based on the similarities ( Figure 7) shows that IsoCleft was able to identify similar arrangement of conserved residues able to bind the common scaffolds of ATP and RFL. Crystal structure 2bl4 represents a group III iron-activated dehydrogenase (alcohol dehydrogenase-PF00465). This protein binds NAD, a common co-factor that shares a common scaffold with ATP. Again the superimposition of the ligands, as seen in the Jmol window ( Figure 6), confirms that the similarities identified relate to residues that evolved to bind common moieties.

These results clearly show that IsoCleft Finder has the ability to predict potential ligands by identifying similarities across domains of dissimilar folds. The potential for detecting binding-site similarities across fold families is clearly interesting from a rational drug design point of view. Such similarities can be used to determine potential cross-reactivity or polypharmacological targets to be integrated into the drug design process. In this scenario detected similarities can determine what kind of potential interactions should be prevented or prioritized in the case of cross-reactivity or polypharmacology respectively.

### Functional predictions

The core engine behind the IsoCleft Finder tool, the IsoCleft program, was previously tested for the detection of binding-site similarities across non-homologous protein families ^8^. IsoCleft has also been applied to study the similarities within histone methyltransferase cofactor binding-sites ^25^ as well as within the human cytosolic sulfotransferase family ^26, 27^. In this section we present four functional predictions performed with IsoCleft Finder to demonstrate its applicability in real scenarios for the prediction of protein function from structure.

The first case is that of a hypothetical protein (cgd2_2020) from *Cryptosporidium parvum* (PDB code 2pd0), solved by the Structural Genomics Consortium (SGC). It is worth noting that this example is one for which all methods currently available as part of the Profunc meta-server for function prediction ^28^ did not provide functional clues (data not shown). These include, among others, global structural (fold) similarity, various sequence and sequence-profile based similarity methods, reverse templates and genome co-localization.

**Figure 8.**
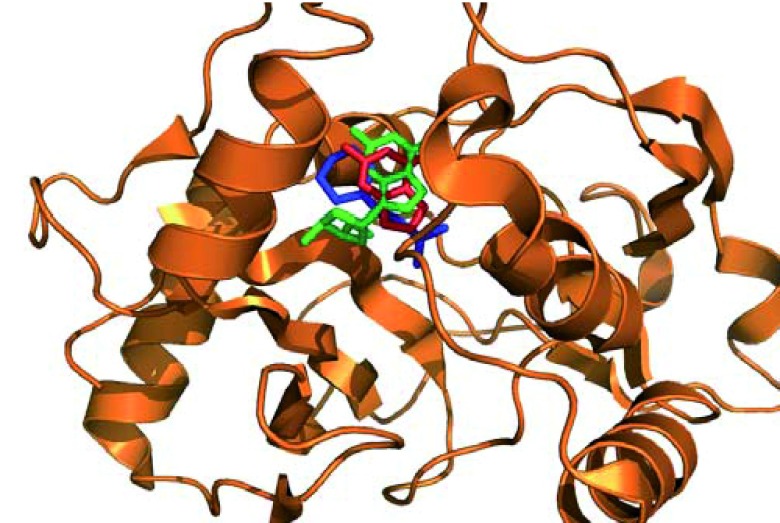
Target ligands superimposed on the query protein structure. The query structure 2pd0 (in orange) is used as reference in which the target binding site ligands guanine (GUN; from PDB entry 1v2h in red) and 5-(6-Amino-2-Fluoro-Purin-9-Yl)-2-Hydroxymethyl-Tetrahydro-Furan-3-Ol [2-Fluoro-2'-Deoxyadenosine] (2FD; from PDB entry 1pke, in green) are superimposed based on their binding site similarities to the query cleft defined by the bound ligand 2-(N-Morpholino)-Ethanesulfonic acid (MES, in blue). A good quality superimposition of the aromatic rings of the different ligands is obtained as a consequence of the superimposition of the binding sites performed by IsoCleft.

While the bound 2-(n-morpholino)-ethanesulfonic acid (MES) is part of the crystallization buffer, it may have serendipitously found its niche in a biologically relevant binding site. For that reason, we used the bound MES to define the binding site. The top two results obtained with IsoCleft Finder are the human purine nucleoside phosphorylase [PDB code 1v2h bound to guanine (GUN; cognate similarity of 0.81) with 21 atoms in common, Tanimoto Score of Similarity 0.404, Z-score 4.10 and p-value 9.20E-03] and the *E. coli* homologue of the same protein [PDB code 1pke bound to 5-(6-amino-2-fluoro-purin-9-yl)-2-hydroxymethyl-tetrahydro-furan-3-ol [2-fluoro-2'-deoxyadenosine] (2FD; cognate similarity of 0.85) with 22 atoms in common, Tanimoto Score of Similarity 0.386, Z-score 3.78 and p-value 1.38E-02. The atoms in common between the 2pd0 cleft and those in 1v2h and 2pke superimpose with RMSD of 1.75 Å and 2.08 Å respectively. Figure 8 shows the target ligands superimposed on the query protein based on the superimposition of the atoms in common. While the superimposition is performed using the detected binding-site atoms in common, the purine rings of the target ligands superimpose quite well with each other as well as with the aromatic ring of the solvent molecule. The fact that the two top hits represent the same reaction in two different organisms ( *Homo sapiens* and *E. coli*) gives further support to the functional prediction.

**Figure 9.**
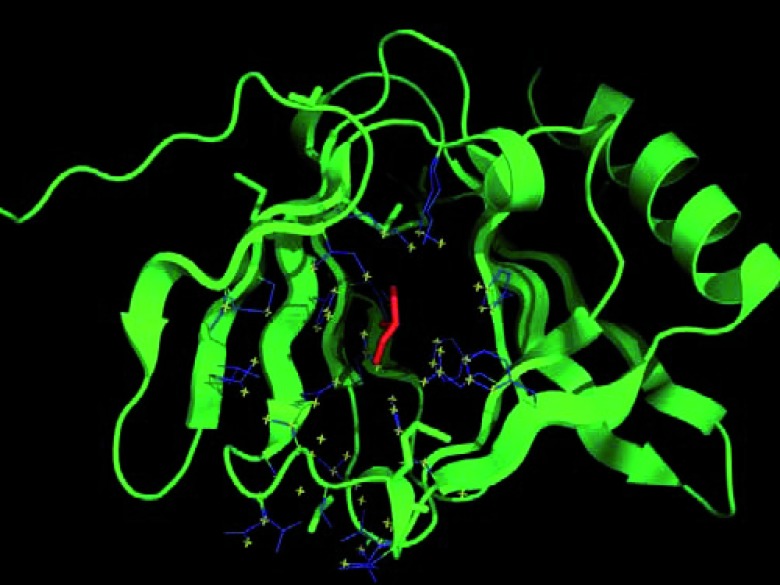
Binding site definition in PDB entry 3d0j. The ligand GOL142A- (in red) is used to define the binding-site (in blue) with the subset of atoms in its surface (in yellow) used as query against the ICFDB subset with cognate similarity level equal to 0.9.

The second example is that of the conserved protein of unknown function ca_c3497 from *Clostridium acetobutylicum* atcc 824, whose structure was recently solved by the Midwest Center for Structural Genomics (MCSG) with PDB code 3d0j. As in the previous example, the Profunc server failed to give any statistically significant functional clues. Again in this case, a molecule from the crystallization buffer (glycerol, ligand ID: GOL142A-) seems to have serendipitously detected the binding-site ( Figure 9). In this case when looking at the top hits based on Tanimoto Score of Similarity (TSS) we find D-glucose (GLC, TSS Z-score 3.74) and D-Glucose-1-Phosphate (G1P, TSS Z-score 3.44) bound to two proteins from different Pfam families (PDB codes 1k1w and 1nt4 respectively). Alternatively, when looking at number of atoms in common we find 27 atoms in common on the top two hits (Z-score 3.17), again to two different Pfam families (PDB codes 1vh3 and 2vfz) bound to two different molecules both containing sugar a moiety in similar positions in space (see http://goo.gl/7QhjD). Taken together, the IsoCleft Finder results suggest a function related to binding sugar moieties.

The third and fourth examples show the results of IsoCleft Finder from our collaboration with the Joint Center for Structural Genomics that were used to support the functional annotation of the proteins in question. The third example is that of the binding-site of the first structure of a member of Pfam family PF06474, a new fold of unknown function from *Pseudomonas aeruginosa*. The IsoCleft Finder analysis identified similarities between the hydrophobic groove along the cleft entrance and dimerization interface of the query protein (PDB code 2h1t), and the lipid-binding site in *Candida rugosa* lipase (PDB code 1lpn; 31 atoms in common, Tanimoto Score of Similarity 0.200, Z-score 3.98, p-value 1.08E-02) suggesting involvement in glycolipid metabolism ^29^.

Fourth, also in collaboration with the Joint Center for Structural Genomics, is the case of two proteins (the product of genes SP00140 and Sbal_2486), the first representatives of PFAM family PF06938 (DUF 1285) from *Silicibacter pomeroyi* (PDB code 2re3) and *Shewanella baltica* (PDB code 2ra9), respectively. The IsoCleft Finder analysis of 2re3 ( Table 2) identified shared features between the inter-domain cleft of 2re3 and sugar, phosphate and purine-binding proteins (PDB codes 1pwh, 1dqa, 1dm3, 1gpe, 1v0j, 1hwy, 2vfs and 1q6p) ^30^.

Similar hits (adenosylcobalamin, heme, dideoxy sugars, NAD, thiamine diphosphate) were obtained for 2ra9 ( Table 3). These similarities, suggest that a nucleotide-based ligand may bind these proteins ^30^ with a possible involvement in signal transduction.

The independent assignment of similar potential ligands to distinct members of the family adds strength to each individual assignment and contributes to the elucidation of the function of the family as a whole.

## Conclusions

In recent years, with the advent of structural genomics projects, a number of proteins with known structure but without functional annotation came to light. In these cases, the detection of binding-site similarities may provide useful functional information complementary to those of existing methods.

In this work we introduce the IsoCleft Finder. IsoCleft Finder is a web-tool for the detection of binding-site similarities between putative ligand-interacting clefts and an associated dataset of target binding-sites with defined levels of cognate-ligand similarity. The ICFDB dataset v.1.6 is useful to study ligand-protein interactions and we encourage its download and use. IsoCleft Finder offers a powerful web-interface to define clefts and to visualize and analyze the obtained results of binding-site similarities.

We demonstrate the use of IsoCleft Finder with cases for which other methods did not provide functional clues. As such, these predictions remain to be validated experimentally. However, in all cases, multiple independent hits obtained with IsoCleft Finder point to similar ligands.

IsoCleft Finder has other applications, such as the detection of potential cross-reactivity targets and small-molecule binders. In the first case, binding-site similarities to unrelated proteins might indicate potential unexpected cross-reactivity targets that would not have been detected otherwise. In the second case, clefts derived from high quality homology models may be used as input to detect potential binding small-molecules that may help produce protein crystals (of the complex) for proteins that would not crystallize in the absence of a stabilizing ligand.

IsoCleft Finder gives access to a broad range of users, including experimental groups without *in house* computational expertise as well as bioinformatics groups without the necessary resources to perform the computationally intensive calculations involved in the detection of binding-site chemical and structural similarities.
