# Supplementary material for: IsoCleft Finder – a web-based tool for the detection and analysis of protein binding-site geometric and chemical similarities
Source: F1000Res. 2013 May 2;2:117. Originally published 2013 Apr 30. [Version 2] doi: 10.12688/f1000research.2-117.v2 (PMC3892921; doi:10.12688/f1000research.2-117.v2)
Supplement: Peer review history

# Peer review: Referee response for version 2

**Published:** 2013-07-18

**Authors:** John Overington

**Affiliations:** 1 Computational Chemical Biology, EMBL European Bioinformatics Institute, Hinxton, Cambridge, UK

The prediction of function is a major challenge from the output of structural genomics programs, and the ability to do this via binding site similarities is an obvious and potentially powerful way to assign function. A further application of protein site similarity approaches is in the area of polypharmacology - the binding of drugs to multiple targets, especially those involving anticipation of adverse drug reactions (ADRs) - a nice example of this sort of similarity is in the recent reporting of the molecular target for the side effects of many sulfa drugs reported by Haruki *et al. *(2013). 

As such, IsoCleft Finder is a potentially useful tool for a range of different researchers. The method focusses on the analysis and prediction of small molecule binding, and as such would miss prediction of protein-protein interactions, and we know that much of biology and disease is composed of protein-peptide and protein-protein interactions. The method in the article is clearly explained, in almost enough detail to allow reimplementation, and there are improvements into the computational efficiency of IsoCleft Finder - the two stage filtering, and the approximate Bron and Kerbosch heuristics. I have a few comments that to me were not clear, or are worthy of clarification:

- How were protonation states and tautomers treated in the analysis? These will have key roles in defining the similarity of the site features.
-  How does the method deal with the possibility of chiraly related sites? The distances between features will be the same, but in real space they will be different. In practice, the real space fit of Ca positions will screen these out, but there could be a set of interesting similarities that are overlooked?
- Can atoms belong to multiple types - hydrophilic, hydrogen bond acceptor, etc? To me it sounds like they could, and if so, what are the equivalence sets, or the priority rules to assign the most important features?
- How does the method score similar sites - same ligand, different asymmetric unit, same ligand orthologous protein, same ligand, different crystal form etc? Is the method stable enough, or are the differences seen enough to calibrate the likelihood of more distant relationships being identified being real?
- Can the method deal with cases where the 'target' is actually composed of a protein and group like a haem? Many enzymes have cofactors, and are obligated to correct site formation. Many methods struggle to handle this simply, so if IsoCleft Finder could handle these it would be a significant improvement over other methods.
- MES doesn't have an aromatic ring, whereas the purine rings do - in the cgd2_2020 case. Fixing the typo here to just reflect ring overlap would fix this.

I have read this submission. I believe that I have an appropriate level of expertise to confirm that it is of an acceptable scientific standard.

# Peer review: Referee response for version 2

**Published:** 2013-06-07

**Authors:** Andras Fiser

**Affiliations:** 1 Albert Einstein College of Medicine , Yeshiva University, Bronx, NY, USA

A web interface is presented to a protein structure based small molecule binding site prediction algorithm that was published before.The algorithm is based on matching binding sites alone with graph matching tools using an annotated set of 7339 structures. The actual method was published before and tested in various conditions. Here, a nicely designed interface is provided that works well. I would like to see more options on the JMOL interface, options to switch between different molecular presentations, as the wire presentation is hard to follow in more complicated instances.

My major reservation is that certain parts, sentences and sometimes almost full paragraphs are identical to those in the authors’ earlier published paper in Bioinformatics that describes the method. Although the authors do mention that they are reiterating the description of the Isocleft program, these sections should be rewritten. It might be better if the current paper focused on the web interface and functionalities and did not try to repeat all the methodological details.

I have read this submission. I believe that I have an appropriate level of expertise to confirm that it is of an acceptable scientific standard, however I have significant reservations, as outlined above.

# Peer review: Referee response for version 2

**Published:** 2013-05-13

**Authors:** Andrew J Bordner

**Affiliations:** 1 Mayo Clinic, Scottsdale, AZ, USA

The IsoCleft Finder web server described in this article can detect and align ligand binding sites in protein structures sharing similar chemical and geometrical properties to the query, even if they do not share homology that is detectable by sequence similarity. The server also provides p-values, which are useful for assessing the confidence of each prediction. The authors discuss a timely application of their method, predicting cognate ligands for the many functionally uncharacterized proteins with experimental structures solved by structural genomics projects. Based on some examples that I ran, the web server returns the results quickly (less than 1 minute). Overall, this paper clearly describes the IsoCleft method and web interface, which should be a useful tool for structural biologists.

A few minor corrections/comments:

- The ICFDB data set contains some small non-biological ligands, like SO _4_ and glycerol, which may bind in ligand binding pockets but not share chemical similarity with the cognate ligand. There should be a brief discussion of why these were included.
- The web server results page currently shows an all-atom wire representation of the entire protein, which makes it difficult to find one’s way around the molecule. I would recommend using a simpler cartoon representation of the protein while keeping the wire representation of site residues
- There is a typo “obtainer” on page 7.

I have read this submission. I believe that I have an appropriate level of expertise to confirm that it is of an acceptable scientific standard.
